# Cohort profile: The Canadian HIV Women’s Sexual and Reproductive Health Cohort Study (CHIWOS)

**DOI:** 10.1371/journal.pone.0184708

**Published:** 2017-09-28

**Authors:** Mona Loutfy, Alexandra de Pokomandy, V. Logan Kennedy, Allison Carter, Nadia O’Brien, Karène Proulx-Boucher, Erin Ding, Johanna Lewis, Valerie Nicholson, Kerrigan Beaver, Saara Greene, Wangari Tharao, Anita Benoit, Danièle Dubuc, Jamie Thomas-Pavanel, Paul Sereda, Shahab Jabbari, Jayson H. Shurgold, Guillaume Colley, Robert S. Hogg, Angela Kaida, on behalf of the CHIWOS Research Team

**Affiliations:** 1 Women’s College Research Institute, Women’s College Hospital, Toronto, Ontario, Canada; 2 Faculty of Medicine, University of Toronto, Toronto, Ontario, Canada; 3 Dalla School of Public Health, University of Toronto, Toronto, Ontario, Canada; 4 Department of Family Medicine, McGill University, Montreal, Quebec, Canada; 5 McGill University Health Centre, Montreal, Quebec, Canada; 6 Faculty of Health Sciences, Simon Fraser University, Burnaby, British Columbia, Canada; 7 BC Centre for Excellence in HIV/AIDS, Vancouver, British Columbia, Canada; 8 Department of Environmental Sciences, York University, Toronto, Ontario, Canada; 9 School of Social Work, McMaster University, Hamilton, Ontario, Canada; 10 Women’s Health in Women’s Hands Community Health Centre, Toronto, Ontario, Canada; 11 Contagion Consulting Group, Vancouver, British Columbia, Canada; Public Health Agency of Canada, CANADA

## Abstract

Globally, women are at increased vulnerability to HIV due to biological, social, structural, and political reasons. Women living with HIV also experience unique issues related to their medical and social healthcare, which makes a clinical care model specific to their needs worthy of exploration. Furthermore, there is a dearth of research specific to women living with HIV. Research for this population has often been narrowly focused on pregnancy-related issues without considering their complex structural inequalities, social roles, and healthcare and biological needs. For these reasons, we have come together, as researchers, clinicians and community members in Canada, to develop the Canadian HIV Women’s Sexual and Reproductive Health Cohort Study (CHIWOS) to investigate the concept of women-centred HIV care (WCHC) and its impact on the overall, HIV, women’s, mental, sexual, and reproductive health outcomes of women living with HIV. Here, we present the CHIWOS cohort profile, which describes the cohort and presents preliminary findings related to perceived WCHC. CHIWOS is a prospective, observational cohort study of women living with HIV in British Columbia (BC), Ontario, and Quebec. Two additional Canadian provinces, Saskatchewan and Manitoba, will join the cohort in 2018. Using community-based research principles, CHIWOS engages women living with HIV throughout the entire research process meeting the requirements of the ‘Greater Involvement of People living with HIV/AIDS’. Study data are collected through an interviewer-administered questionnaire that uses a web-based platform. From August 2013 to May 2015, a total of 1422 women living with HIV in BC, Ontario, and Quebec were enrolled and completed the baseline visit. Follow-up interviews are being conducted at 18-month intervals. Of the 1422 participants at baseline, 356 were from BC (25%), 713 from Ontario (50%), 353 from Quebec (25%). The median age of the participants at baseline was 43 years (range, 16–74). 22% identified as Indigenous, 30% as African, Caribbean or Black, 41% as Caucasian/White, and 7% as other ethnicities. Overall, 83% of women were taking antiretroviral therapy at the time of the baseline interview and of them, 87% reported an undetectable viral load. Of the 1326 women who received HIV medical care in the previous year and responded to corresponding questions, 57% (95% CI: 54%-60%) perceived that the care they received from their primary HIV doctor had been women-centred. There were provincial and age differences among women who indicated that they received WCHC versus not; women from BC or Ontario were more likely to report WCHC compared to participants in Quebec. They were also more likely to be younger. CHIWOS will be an important tool to develop care models specific for women living with HIV. Moreover, CHIWOS is collecting extensive information on socio-demographics, social determinants of health, psychological factors, and sexual and reproductive health and offers an important platform to answer many relevant research questions for and with women living with HIV. Information on the cohort can be found on the study website (http://www.chiwos.ca).

## Introduction

Life expectancy and quality of life for people with HIV in Canada have rapidly improved due to the successes of antiretroviral therapy (ART) and improved HIV care [1–3]. However, dampening these achievements is a persistent gender gap in access to, retention in, and quality of care that favours men in the Canadian context despite a steady representation of women in the HIV-positive population [4, 5]. The Public Health Agency of Canada estimated that the number of people with HIV at the end of 2014 was 75 500, of which 16 880 were women [6]. The number of positive HIV tests attributed to women in Canada has increased since the beginning of the epidemic with 23% of new infections occurring in women in 2014. While the proportion of all incident HIV cases occurring in women has stabilized at approximately one quarter, it is noted to be a concern that it has not decreased [6]. Globally, biological, social, structural, and political factors intersect to increase women’s vulnerability to HIV. In Canada, and many high-income countries, these inequities subsequently impact women’s experiences after HIV diagnosis; a phenomenon known as the feminization of HIV [5, 7–9]. This differs from the HIV gender gap in countries throughout sub-Saharan Africa [10–12], where women have been found to more readily engaged in care in some regions due to efforts focused within Maternal and Child Health Programs. However, this finding also highlights the 'gendered' vulnerability of HIV for women and how this calls for attention at national and regional levels for women-specific HIV services in Canada. In Canada, women’s vulnerability is the result of the complex intersection of gender with other dimensions of identity in systems where racism, colonial legacies, homophobia, transphobia, heterosexism, and sexism are inherent [13–15]. As such, Indigenous women, women from or who partner with men from HIV-endemic countries, women who use or used drugs, women involved in sex work, young women, and trans women are particularly vulnerable to HIV and constitute the majority of women with HIV in Canada [6,15].

Women with HIV also encounter added obstacles in healthcare in a clinical model that does not recognize their gendered experiences of HIV. Women with HIV frequently report worse clinical outcomes than men, including higher rates of viral rebound [16], lower quality of care [17] and inattention to their health and social needs [18]. Perpetuating this clinical insufficiency is the longstanding lack of research specific to women with HIV [19, 20]. When available, research for women with HIV has often been narrowly focused on pregnancy-related issues without considering their broader health issues [19]. Given the distinct ways that women with HIV are situated with respect to structural inequalities, social roles, biological needs, and healthcare complexities, it is crucial to address women with HIV’s specific health needs. As such, we have come together to develop and investigate the impact of a model of care called ‘women-centred HIV care (WCHC)’ in addressing these inequities. Recently, the World Health Organization (WHO) released a consolidated guideline on the sexual and reproductive health and rights of women living with HIV [21]. This WHO guideline calls for women-centred care [21], however, to date, little is known about this care model.

The Canadian HIV Women’s Sexual and Reproductive Health Cohort Study (CHIWOS), was created by, with, and for women with HIV in collaboration with academic researchers, clinicians, and community partners in response to calls from women with HIV for increased research focusing on the lives and care of women with HIV [22]. This cohort was developed to longitudinally investigate the concept of WCHC and its impact on the overall (quality of life), HIV (e.g., ART use, viral suppression), women’s (e.g., cervical cancer screening), mental (e.g., depression), sexual (e.g., sexual functioning and satisfaction), and reproductive (e.g., contraceptive use, pregnancy) health outcomes of women with HIV. Through a literature review and focus groups with women with HIV, an initial definition of WCHC was created: *“care that supports women living with HIV to achieve the best health and wellbeing as defined by them*. *This type of care recognizes*, *respects*, *and addresses women’s unique health and social concerns*, *and recognizes that they are connected*. *Because this care is driven by women’s diverse experiences*, *it is flexible and takes their different needs into consideration”*. The purpose of this paper is to describe our methodology and present the cohort and preliminary findings related to perceived WCHC.

## Material & methods

### Study guiding frameworks

Crucial to the functioning of the project, CHIWOS operates using a community-based research (CBR) approach [23], with the Greater Involvement of People Living with HIV (GIPA) [24] at its centre and follows the theoretical approaches of critical feminism, anti-oppression and intersectionality [25, 26]. Women with HIV have been involved with the project from the beginning and at every stage, and have been hired and trained in research conduction, as peer research associates (PRAs). The operationalization of CBR in this study is reviewed in detail elsewhere [27].

### Study setting and population

**Fig 1:**
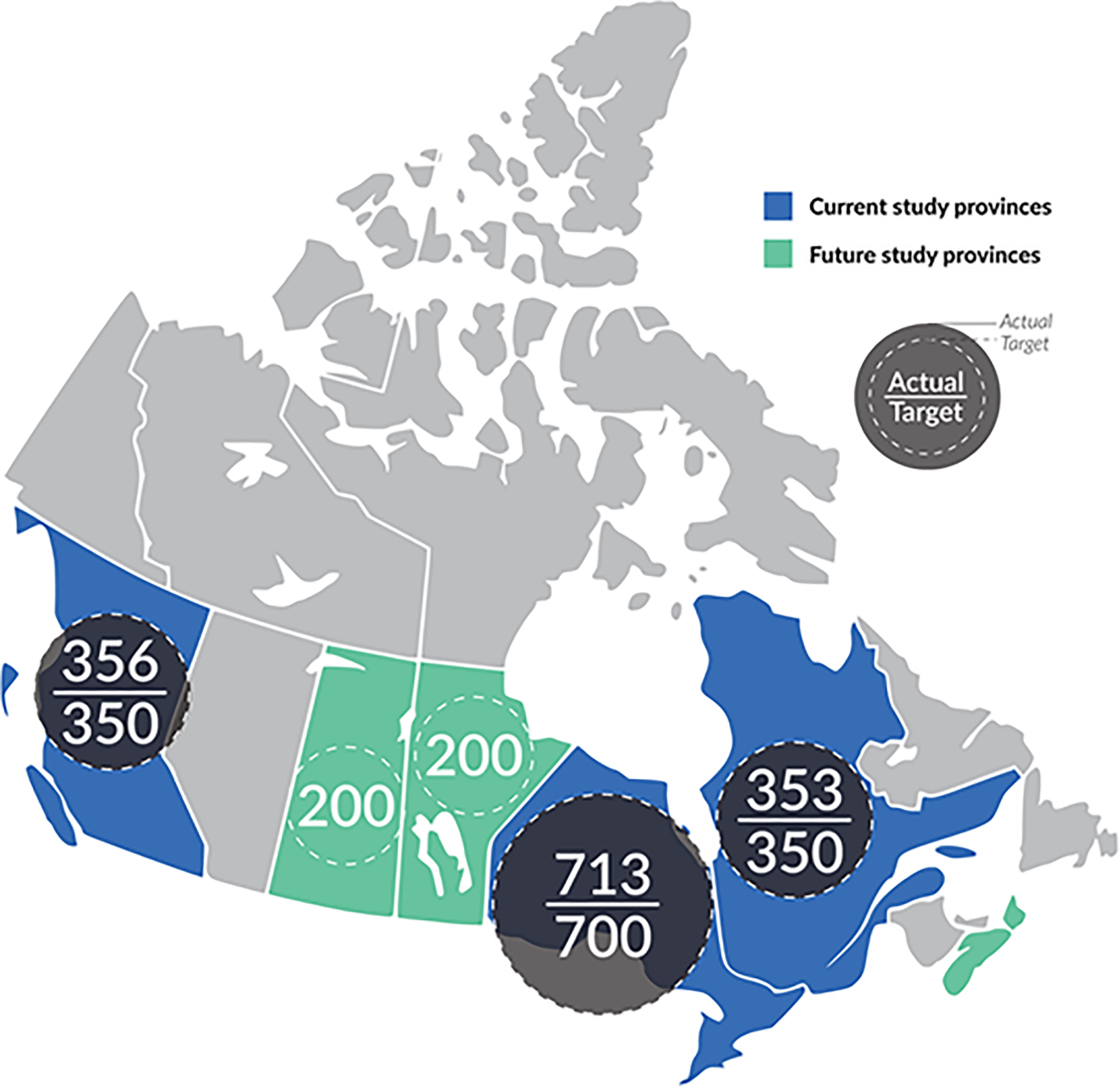
CHIWOS provinces with target and actual enrolment numbers. Current Canadian HIV Women’s Sexual and Reproductive Cohort Study (CHIWOS) sites (in blue)–i.e. British Columbia, Ontario, and Quebec, and upcoming sites (in green)–i.e. Saskatchewan and Manitoba. Also shown are the target (dotted circles) and actual (dark circles) recruitment numbers per province.

CHIWOS is currently being conducted in the three Canadian provinces of British Columbia (BC), Ontario (ON), and Quebec (QC) (Fig 1). These three provinces were initially selected because of the high percentage of women with HIV in Canada that would be captured in these study provinces (82%) [6]. Two additional Canadian provinces, Saskatchewan and Manitoba, are joining the cohort in 2018.

Eligible participants self-identified as: women (including cis, trans, intersex, two-spirit and gender queer or questioning people who identified as women); being 16 years of age or older; being diagnosed with HIV; and living in one of the study provinces at the time of the baseline visit.

### Study sampling and recruitment

The study applied a non-random, purposive sampling frame. Women with HIV were geographically enrolled based on the distribution of women with HIV in each provincial region as found in provincial public health reports [28, 29, 30]. Our sample size target was 350 women from BC, 700 women from ON, and 350 from QC, which allowed us to detect a 50% probability of a binary outcome at the provincial level, with +/- 10% margin of error, and a 90% confidence interval.

Our purposive sampling also aimed to enrol women who were potentially harder-to-reach and underserved to enable analysis regarding the healthcare access and needs, and health outcomes of a group of women often left out of research [31]. The groups of women defined as harder-to-reach were determined by investigators’ clinical expertise and difficulty in enrolling these populations into other studies [32]. The groups of women included as harder-to-reach are described in a supplemental Table and included trans women, Indigenous women, women who inject drugs and young women (< 30 years of age) (S1 Table).

Recruitment occurred from August 2013 to May 2015 through: 1) personal networks of and word-of-mouth by the PRAs and other women with HIV; 2) community-based and AIDS service organizations; 3) HIV clinics; 4) online through our website, Facebook page, and Twitter presence; 5) our provincial community advisory board members; and 5) posters and flyers posted in non-HIV-specific community settings where women attend such as women’s shelters.

### Study procedures

Primary ethics approval was obtained from Women’s College Hospital (ON), Simon Fraser University (BC), University of British Columbia/Providence Health (BC), and McGill University Health Centre (QC) from their respective Research Ethics Boards (REBs). Study sites with independent REBs obtained their own approval prior to commencing enrolment.

Potential participants were screened by a trained PRA or the provincial coordinator. If they met the inclusion criteria, they were then provided with the informed consent to participate. After consenting, participants were asked to complete a PRA-administered web-based questionnaire, programmed using White Label FluidSurveys™ data capture software. Whenever possible, the interviewer-administered survey was carried out in person but when required was done by phone or Skype. The online programming included skip patterns and response validation towards maximizing data quality and providing ‘real time’ data capture. Surveys could be completed in English or French. For women who did not speak either language, the survey could be completed with the assistance of a translator. The median length of time to complete the baseline survey was 120 minutes [interquartile range (IQR): 90,150]. The lengthy survey completion time was vetted by the PRAs and deemed acceptable as important topics often left out of other research projects were included (e.g. violence and sexual health).

Follow-up visits are occurring at 18-month intervals. Eighteen-month follow-up was chosen so as to not over-burden women with research participation while allowing enough time to pass for changes to occur. An 18-month interval was also selected in an attempt to minimize recall bias and risks of loss-to-follow-up (LTFU).

Visit 2 questionnaires began June 23, 2015 and finished January 31, 2017. Follow-up was carried out by the same PRA (when possible) by contacting the participants by phone or email depending on the preferred means indicated by the participant. In order to maximize retention, follow up interviews were permitted beyond the 18-month window so long as they were completed before official closure of the follow up period. A follow-up procedure was developed including three attempts by the PRA, followed by contacting the community-based organization if one was involved in the initial recruitment. If not, re-contacting through the participant’s clinic was sought as long as the clinic had REB approval. Several additional methods were also used to minimize LTFU including an online presence and having close partnerships with community-based organizations. Visit 3 questionnaires began February 1, 2017.

### Questionnaires and study variables

The baseline CHIWOS questionnaire collected extensive data on demographics, social determinants of health, HIV clinical outcomes, use of WCHC, health and social services use, psychological and emotional health, sexual and reproductive health, substance use, and experiences of violence, stigma, and discrimination [33]. The survey contained 436 questions and 2136 variables; however, participants completed only those questions relevant to their identity and experience. As the survey was the only means of data collection, all variables are self-reported including clinical variables such as viral load (VL) and hepatitis status. An extensive CBR approach to survey development was used and is described elsewhere [34]. The survey development team used validated scales when available. The final baseline questionnaire included nine sections that are presented in Table 1. Detailed descriptions of the themes covered and validated scales used in each questionnaire section are presented in S2 Table.

**Table 1:** CHIWOS questionnaire sections.

Section	Section Topic
SECTION 1	Demographics and Socio-economic Status
SECTION 2	Medical and HIV Disease Information
SECTION 3	Health Care and Support Service Utilization
SECTION 4	Women’s Reproductive Health
SECTION 5	Stigma and Discrimination
SECTION 6	Substance Use
SECTION 7	Violence and Abuse
SECTION 8	Women's Sexual Health
SECTION 9	Emotional Wellbeing, Resiliency, and Health Related Quality of Life

Every question had the options of “don’t know” and “prefer not to answer” for the participant to answer. An answer was required for each question to move on to the next page. Sections 7 and 8 on Violence and Abuse and Women’s Sexual Health could be self-administered or declined due to the sensitivity of the topics and the risk of triggering.

### Primary variable of interest

The primary variable of interest is the concept of WCHC that is measured in Section 3 with a scale that we developed based on our literature review and focus groups [5, 31]. We also developed a brief scale to assess overall perceived WCHC of the women’s HIV doctor and clinic. Perceived WCHC of one’s HIV clinic or doctor was assessed using a 5-point Likert scale measuring agreement with statements such as “Overall, I think that the care I have received from my HIV clinic (or doctor) has been women-centred”. Responses were categorized into: Strongly Agree/Agree (‘Perceived WCHC') vs. Neutral (‘Neutral WCHC’) vs. Strongly Disagree/Disagree (‘No perceived WCHC’). Our definition of WCHC was provided to participants prior to asking the set of WCHC questions.

### Validity and test-retest reliability

An *a priori* strategy for ensuring and determining the baseline questionnaire’s validity and reliability was developed [22]. Test-rest reliability of the questionnaire measures was assessed among 30 participants (10 per province) completing the baseline questionnaire twice, separated by approximately 2 weeks [35]. The Kappa statistic and the intraclass correlation coefficient were used to assess reliability of categorical variables and continuous variables, respectively. We used the following cut-offs to interpret the strength of agreement for the Kappa coefficient: ≤0 = poor, .01–.20 = slight, .21–.40 = fair, .41–.60 = moderate, .61–.80 = substantial, and .81–1 = almost perfect.

### Statistical analyses

The sociodemographic, clinical and WCHC variables were determined for the overall population and by province using frequencies and proportions for categorical variables and medians and either ranges or IQRs for continuous variables. Comparisons were made between provinces using the Chi-square test for categorical variables and the Kruskal-Wallis test for continuous variables.

### Potential linkage to other data sources

CHIWOS has been designed to allow for data linkages to existing provincial and national administrative and research datasets, including the Drug Treatment Program (DTP) in BC, the Institute for Clinical Evaluative Sciences (ICES) population-level data and the Ontario HIV Treatment Network Cohort Study (OCS) [36] in ON, and the Montreal HIV patient database of the AIDS Network of Fonds de Recherche du Québec-Santé (FRQS) and the Régie de l'assurance maladie du Québec (RAMQ) in QC. In addition, CHIWOS is affiliated with the Canadian Observational Cohort Collaboration (CANOC) [37, 38]. Linkage to administrative datasets allows for the validation of self-reported clinical and laboratory responses (e.g. VLs, CD4+ count and ART use). Also, such linkages could allow the merging and analyses of an administrative dataset with the CHIWOS dataset, which is rich in psychosocial and social determinant variables.

## Results

### Study population

As is seen in Table 2, CHIWOS has successfully enrolled a diverse cohort of 1422 women with HIV (356 from BC [25%], 713 from ON [50%], 353 from QC [25%]). Baseline demographics are presented in Table 2. The median age of the participants was 43 years (range, 16–74). Participants represented diverse communities: 22% identified as Indigenous, 30% as African, Caribbean, or Black, 41% as Caucasian/White, and 7% as other ethnicities. The CHIWOS cohort successfully enrolled harder-to-reach or underserved communities of women with 31% and 6% reporting injection drug use history and current sex work, respectively. Overall, 83% of women were currently taking ART and 87% of them reporting an undetectable VL.

**Table 2:** Baseline characteristics of study participants overall and by province.

Demographic Characteristics	N with	Total	British Columbia	Ontario	Quebec	p-value
	responses	*N = 1422*	*N = 356*	*N = 713*	*N = 353*	
						
**Median Age (IQR)**	1422	43(36–51)	44(37–51)	41(34–49)	46(38–53)	<0.001
**Gender identity**	1422					
Woman		1359 (96%)	342 (96%)	679 (95%)	338 (96%)	0.804
Trans woman/Two-spirit/Queer/Intersex/Other		63 (4%)	14 (4%)	34 (5%)	15 (4%)	
**Sexual orientation**	1417					
Heterosexual		1237 (87%)	294 (83%)	617 (87%)	326 (92%)	<0.001
LBQQ2S		180 (13%)	61 (17%)	92 (13%)	27 (8%)	
**Ethnicity**	1422					
Indigenous–First Nations, Métis or Inuit		318 (22%)	161 (45%)	149 (21%)	8 (2%)	<0.001
African/Caribbean/Black		418 (30%)	28 (8%)	227 (32%)	163 (46%)	
Caucasian/White		585 (41%)	139 (39%)	280 (39%)	165 (47%)	
Other*		103 (7%)	28 (8%)	57 (8%)	17 (5%)	
**Ever incarcerated**	1420	524 (37%)	222 (62%)	205 (29%)	97 (28%)	<0.001
**Injection drug use history**	1396	439 (31%)	225 (63%)	132 (19%)	83 (24%)	<0.001
**Involved in sex work**	1422					<0.001
Yes		82 (6%)	36 (10%)	30 (4%)	16 (5%)	
No		1225 (86%)	300 (84%)	614 (86%)	311 (88%)	
Don’t’ know/Prefer not to answer		115 (8%)	20 (6%)	69 (10%)	26 (7%)	
**Clinical Characteristics**						
			
**HCV co-infection**	1415	451 (32%)	201 (56%)	147 (21%)	103 (29%)	<0.001
**HBV co-infection**	1405	119 (8%)	48 (13%)	35 (5%)	36 (10%)	<0.001
**Median years living with HIV (IQR)**	1374	11 (6–17)	12 (7–18)	10 (5–15)	13 (8–18)	<0.001
**Received HIV medical care in last year**	1420	1330 (94%)	350 (98%)	641 (90%)	339 (96%)	<0.001
**Currently taking ART**	1415	1175 (83%)	318 (89%)	534 (75%)	323 (92%)	<0.001
**Undetectable viral load (self-report)**^**#**^	1377					<0.001
Undetectable (below 50 c/mL)		1099 (80%)	286 (82%)	503 (74%)	308 (88%)	
Detectable (over 50 c/mL)		204 (15%)	51 (14%)	122 (18%)	31 (9%)	
Don’t’ know/Prefer not to answer		76 (5%)	13 (4%)	52 (8%)	11 (3%)	
**Most recent CD4 (self-report)**	1382					
<200 cells/mm3		75 (5%)	30 (9%)	22 (3%)	23 (6%)	<0.001
200–500 cells/mm3		386 (28%)	114 (32%)	173 (26%)	99 (28%)	
>500 cells/mm3		698 (51%)	166 (47%)	363 (53%)	169 (48%)	
Don’t’ know/Prefer not to answer		223 (16%)	42 (12%)	122 (18%)	59 (17%)	

IQR, interquartile range; LBQQ2S, lesbian, bisexual, queer, questioning, or two-spirit; HCV, hepatitis C; HBV, hepatitis B; ART, antiretroviral therapy.

*Other ethnicities included Chinese/Filipino/Japanese/Korean/Latin America/South Asian/Southeast Asian/Arab/West Asian/Multiple ethnicities.

^#^80% (1097/1377) of the overall cohort self-reported having an undetectable viral load; of the 1175/1415 women on ART, 87% (1025/1175) had an undetectable viral load.

Refusal data were collected qualitatively from PRAs during the baseline enrolment period. As per the PRAs, the reasons for not enrolling were infrequently due to refusal, but tended to be due to practical issues, personal health concerns, and difficulty re-connecting with a potential participant. In addition, some women who were screened were not enrolled due to stratified sampling targets to enroll women from under-represented priority populations.

As all variables were self-reported, attempts are underway to validate them compared to objective ones such as laboratory data. Thus far, we have compared the self-reported VLs with laboratory-confirmed VL data from participants in BC and found a high degree of validity of self-report [39]. BC is the only study province where linkage to clinical data is possible as the DTP database held at the BC Centre for Excellence in HIV/AIDS is a population-based registry capturing 100% of laboratory VL data in BC. The survey VL data were linked to the clinical DTP data for 99% of participants (n = 355 of 356); only one participant remained unlinked and 19 were excluded due to missing self-reported or lab data. The positive predictive value was 94 [95% confidence interval (CI): 90–96] and the negative predictive value was 80 (95% CI: 67–90).

### Self-reported perceived women-centred HIV care (WCHC)

Of the 1330 women who received HIV medical care in the previous year (out of 1420 who responded), all reported on corresponding women-centred questions. Of these 1330 women, 61% (95% CI: 58%-63%) perceived that the care they received from their primary HIV doctor or clinic had been women-centred (Table 3).

**Table 3:** Perceived experience of women-centred HIV care of HIV doctor and clinic overall and by province of participants receiving HIV care (N = 1330).

	Total N	Total	British Columbia	Ontario	Quebec	p-value
		*N = 1332*	*N = 350*	*N = 641*	*N = 341*	
**Perceived WCHC of HIV Doctor and/or Clinic**						
				
**Perceive care by HIV doctor to be women-centred**	1326	757 (57%)	232 (67%)	401 (63%)	124 (37%)	<0.001
**Perceive care at HIV clinic to be women-centred**	1323	709 (53%)	214 (61%)	380 (59%)	115 (34%)	<0.001
**Perceive care by HIV doctor and/or clinic to be women-centred**	1329	807 (61%)	243 (69%)	418 (65%)	146 (43%)	<0.001
**Women-centred care is important to me**	1328	1065 (80%)	289 (83%)	524 (82%)	252 (74%)	<0.001
**Satisfaction with care from HIV Doctor and Clinic**						
				
**Satisfied with the care received from HIV doctor**	1329	1228 (92%)	318 (91%)	589 (92%)	321 (95%)	0.167
**Satisfied with the care received from HIV clinic**	1328	1226 (92%)	315 (90%)	591 (92%)	320 (94%)	0.160
**Care satisfaction depends on how women-centred it is**	1323	791 (59%)	204 (58%)	430 (67%)	157 (46%)	<0.001

WCHC, women-centred HIV care.

### Bivariate analyses of perceived WCHC by HIV doctor

Table 4 shows the bivariate analyses of demographic, clinical participant variables, and reporting of WCHC provided by the women’s HIV doctor. It should be noted that although 61% of the total sample perceived that their primary HIV care was women-centred, significant regional differences were reported. While 67% and 63% of women in BC and ON, respectively, reported perceived WCHC, only 37% women reported WCHC in QC. Also, women reporting WCHC were more likely to be younger; however, only by a median of two years.

**Table 4:** Bivariate analysis of characteristics by perceived women-centred HIV care from HIV doctor.

Socio-Demographic Characteristics		N with responses	Perceived WCHC	Neutral WCHC	No perceived WCHC	p-value
		1326	N = 757	N = 271	N = 298	
			n (%)			
**Province**		1326				
British Columbia			232 (67%)	46 (13%)	70 (20%)	<0.001
Ontario			401 (63%)	129 (20%)	111 (17%)	
Quebec			124 (37%)	96 (28%)	117 (35%)	
**Median Age [IQR]**		1326	43 (35–50)	45 (37–53)	45 (38–52)	0.006
**Age categories**		1326				
** <30**			82 (71%)	16 (14%)	18 (15%)	0.007
** 30–50**			476 (58%)	162 (20%)	187 (22%)	
** >50**			199 (52%)	93 (24%)	93 (24%)	
**Gender identity**		1326				
Woman			725 (57%)	260 (20%)	289 (23%)	0.656
Trans woman/Two-spirited/Queer/Other			32 (62%)	11 (21%)	9 (17%)	
**Sexual orientation**		1321				
Heterosexual			664 (57%)	236 (20%)	260 (23%)	0.983
LBQQ2S			91 (57%)	33 (20%)	37 (23%)	
**Ethnicity**		1326				
Indigenous–First Nations, Métis or Inuit			177 (63%)	41 (15%)	62 (22%)	0.022
White/Caucasian			217 (55%)	94 (24%)	80 (21%)	
African/Caribbean/Black			302 (54%)	123 (22%)	132 (24%)	
Other^#^			62 (62%)	13 (13%)	24 (25%)	
**Relationship Status**		1324				0.271
Partnered or married or in a relationship			247 (59%)	79 (19%)	94 (22%)	
Single			372 (58%)	130 (21%)	136 (21%)	
Separated / Divorced / Widowed / Other			136 (51%)	62 (23%)	68 (26%)	
**Ever incarcerated**		1324				
Yes			255 (53%)	90 (19%)	134 (28%)	0.001
No			502 (60%)	180 (21%)	163 (19%)	
**HIV Health Outcomes**						
			
**Median years living with HIV (IQR)**		1285	11 (6–17)	11 (6–16)	12 (7–18)	0.078
**Categories**		1285				
** <5 years**			142 (60%)	47 (20%)	46 (20%)	0.248
** 5–10 years**			195 (59%)	73 (22%)	65 (19%)	
** >10**			397 (55%)	142 (20%)	178 (25%)	
**Hepatitis B**		1311				
Yes			57 (8%)	25 (9%)	31 (11%)	0.274
No			693 (92%)	243 (91%)	262 (89%)	
**Hepatitis C**		1321				
Yes			226 (30%)	79 (29%)	116 (39%)	0.012
No			527 (70%)	191 (71%)	182 (61%)	
**Currently taking ART**		1319				
Currently taking ART			639 (85%)	251 (93%)	256 (86%)	<0.001
Not currently taking ART, but previously			28 (4%)	6 (2%)	21 (7%)	
Never on ART			85 (11%)	13 (5%)	20 (7%)	
**Undetectable viral load (self-report)**		1316				
Undetectable (below 50 c/mL)			600 (80%)	221 (82%)	248 (84%)	0.591
Detectable (over 50 c/mL)			109 (15%)	34 (13%)	37 (13%)	
Don’t know/Prefer not to answer			42 (5%)	14 (5%)	11 (3%)	
**Most recent CD4 count (self-report)**		1321				0.113
<200 cells/mm3			30 (4%)	18 (7%)	22 (7%)	
200–500 cells/mm3			223 (29%)	71 (26%)	76 (26%)	
>500 cells/mm3			397 (53%)	142 (52%)	145 (49%)	
Don’t know/Prefer not to answer			105 (14%)	40 (15%)	53 (18%)	

WCHC, women-centred HIV care; IQR, interquartile range; LBQQ2S, lesbian, bisexual, queer, questioning, or two-spirit; HCV, hepatitis C; HBV, hepatitis B; ART, antiretroviral therapy.

^#^Other ethnicities included Chinese/Filipino/Japanese/Korean/Latin America/South Asian/Southeast Asian/Arab/West Asian/Multiple ethnicities

### Test-retest reliability assessment

Test-rest reliability of the baseline questionnaire measures is presented in Table 5. The majority of the variables of interest scored either “substantial” or “almost perfect”, with some scoring “moderate”. As perceived WCHC by HIV doctor was more reliable than by clinic, we have chosen it to be our primary variable of interest (see Table 4).

**Table 5:** Test-retest reliability of key CHIWOS variables.

Demographic Characteristics	Kappa*/ICC^#^	95% CI of Kappa statistic or ICC	Strength of agreement
Age at interview	1	(1, 1)	Almost Perfect
Gender identity	0.78	(0.37, 1.00)	Substantial
Sexual orientation	1	(1, 1)	Almost Perfect
Ethnicity	0.93	(0.79, 1.00)	Almost Perfect
Ever incarcerated	1	(1, 1)	Almost Perfect
Injection drug use history (ever)	0.96	(0.88, 1.00)	Almost Perfect
**Clinical characteristics**			
HCV co-infection	0.93	(0.79, 1.00)	Almost Perfect
HBV co-infection	0.72	(0.36, 1.00)	Substantial
Median years living with HIV	0.93	(0.86, 0.97)	NA
Received HIV medical care in last year	1	(1, 1)	Almost Perfect
Currently taking ART	1	(1, 1)	Almost Perfect
Undetectable viral load (self-report)	1	(1, 1)	Almost Perfect
Most recent CD4 (self-report)	0.71	(0.41, 1.00)	Substantial
**Perceived women-centred care by HIV Doctor or Clinic**			
Perceive care by HIV doctor to be women-centred	0.67	(0.41, 0.93)	Substantial
Perceive care at HIV clinic to be women-centred	0.6	(0.32, 0.88)	Moderate
Women-centred care is important to me	0.49	(0.16, 0.81)	Moderate
Satisfied with the care received from HIV doctor	0.8	(0.69, 1.00)	Substantial
Satisfied with the care received from HIV clinic	0.86	(0.75, 1.00)	Almost Perfect
Care satisfaction depends on how women-centred it is	0.66	(0.40, 0.93)	Substantial

CHIWOS, Canadian HIV Women’s Sexual and Reproductive Health Cohort Study; CI, confidence intervals; ICC, intraclass correlation coefficient

Note:

*Standard Kappas were calculated for nominal variables, weighted Kappas for ordinal variables and prevalence adjusted Kappa for rare observations.

^#^ICCs were calculated for continuous variables; strength of agreement not available.

### Retention rates for visit 2

The overall retention rate for the study was 88% with 1252 participants (of 1422) having completed visit 2. The provincial retention rates were 84% (299 of 356) in BC, 89% (632 of 713) in ON and 91% (321 of 353) in QC. Of the 170 women who did not complete visit 2, 26 were deceased (12 in BC, 10 in ON and 4 in QC); 22 women withdrew from the study, including 7 who moved to another province and 3 who were palliative. 24 opted out of completing visit 2 (but are potentially interested in completing visit 3), including 5 women who were incarcerated; and we were unable to contact 98 (i.e., LTFU) (31 in BC, 54 in ON and 13 in QC) but outreach efforts will continue for Wave 3.

## Discussion

CHIWOS has greatly contributed to the field of women and HIV by: 1) applying a CBR approach to a large national cohort study, 2) meaningfully applying the GIPA principles, and 3) enrolling harder-to-reach, and underserved communities of women with HIV in Canada. We enrolled 1422 diverse women with HIV from BC, ON and QC and have retained 1252 in our second study visit.

The primary objective of CHIWOS, which is to develop and test the concept of WCHC, is novel. As such, the study team had to develop a new scale used to determine perceived WCHC. Therefore, test-retest reliability was an important step before identifying the best variable for perceived WCHC. Interestingly, we only found moderate test-retest time reliability for perceived WHCH by the clinic. Also, care from a clinic is often provided by multiple people and care providers, which could complicate the conceptualization of WCHC from the clinic as a whole. The fact that these questions were created and being used for the first time may suggest that they were not well understood by the participants in relation to their clinic. Fortunately, the variable of WCHC by HIV doctor had substantially better reliability and was thus used as the primary variable of interest. The team intends to continue to explore the variable of WCHC by clinic to determine if our scale could be altered to better measure this variable.

Preliminary findings suggest some important considerations regarding our primary objective of exploring the concept of WCHC. The results of Visit 1 suggest that self-reported perception of WCHC varies by province with a significant difference between women in BC and ON and their peers in QC. The study team has given this finding substantial consideration to explore possible explanations. For participant in BC, one potential explanation is the highly integrated care that many women with HIV received at the Oak Tree Clinic in Vancouver. As the only centralized, women and family-focused care centre in Canada, the benefit of Oak Tree Clinic may be captured in this result. As for the high rate of self-reported WCHC in ON, the explanation for this finding remains unclear and will continue to be unpacked through the results of Visits 2 and 3, as will the low rates of self-reported WCHC in the province of QC.

Age also appears to be an important construct to evaluate in relation to WCHC with significantly more younger women reporting WCHC. The clinical attention to reproductive health in younger women with HIV may contribute to this finding. However, with an aging population of women with HIV, WCHC will need to broaden and include life-course issues such as menopause and other age-related co-morbidities. Finally, the results presented in Table 4 capture an interesting finding that ART-naïve participants were significantly more likely to report WCHC, the nuances of this finding are hard to interpret given the small sample size but will continue to be explored throughout the course of this longitudinal cohort.

### Strengths and weaknesses

Having enrolled 1422 women with HIV, CHIWOS is the largest Canadian cohort study of women with HIV and will contribute data to our understanding of the current state of care and wellbeing of women with HIV in Canada. An acknowledged limitation of CHIWOS is that the cohort is not a random sample and may not be statistically representative of the wider population of women with HIV in Canada. Having also used purposive selection to enrol marginalized women, there is the potential for selection bias in those marginalized women who did enrol and thus findings may not be representative of all marginalized women with HIV in Canada. Nonetheless, CHIWOS has enrolled approximately 10% of all women with HIV in Canada and will provide important findings.

An additional concern is the potential for attrition given the relatively long period between visits and the risk of loss-to-follow-up of a harder-to-reach population. In an attempt to mitigate loss-to-follow-up, we have requested multiple means of contacting participants. We have also created a strong study presence online and via social media, and strong connections with clinics and community partners. This has not been an issue between the first and second data collection points.

Self-report may lead to social desirability bias, whereby participants provide answers to questions that they think the interviewer wants to hear, regardless of whether the answer is truthful or not. We have attempted to minimize this and other forms of reporting bias through PRA training, “smart survey” design with definitions for terms used, and the option to complete certain parts of the survey without the interviewer (i.e., sexual health and violence). While HIV clinical data may be poorly reported (e.g., VL), an initial analysis carried out in BC found excellent correlation with confirmed laboratory values [39]. This method of data capture also potentiates limitations in the reporting of clinical variables such as hepatitis C status, in that nuances like whether an infection has spontaneously cleared would not be captured. A general weakness of questionnaire-based studies is missing data. We attempted to mitigate missing data with PRA training and by electronically requiring an answer before moving to the next question. This means that it was impossible to skip a question and thus there is no missing data. “Don’t know” and “prefer not to answer” were choices for every question and can be used as needed. Thus far, overall results have not yielded a high frequency of these responses.

CHIWOS’s use of CBR from inception, prioritizing GIPA and the expertise of women with HIV is a strength of the study and fills a long-standing gap for gender transformative HIV research in Canada. The formative study phase also provided the advantage of knowledge creation using qualitative methods. Developing and finalizing the survey using an extensive process of community-based survey development enabled the needs of various community members and stakeholders to shape the sections and questions [34]. The longitudinal nature of CHIWOS enables the research team to adapt the survey to include questions about areas of emerging priority for women with HIV.

## Conclusion

CHIWOS aims to be a CBR leader in explicating the concept of WCHC among diverse women with HIV living in Canada. It is achieving this aim through carrying out a large bilingual longitudinal cohort study of diverse women with HIV from across Canada, which is led by women with HIV, themselves, in partnership with academic researchers and clinicians. As a group, we are excited to further develop this model of care specific to women with HIV. CHIWOS also provides an opportunity to investigate many social, structural, behavioural, and clinical questions as they relate to diverse groups of women living with HIV across Canada, with results applicable to policy and programming in Canada, and around the world.

## Additional resources

The CHIWOS investigators believe in open access of the operational resources to all interested in CBR and/or HIV women’s health and they can be found on our website: www.chiwos.ca.

Other specific resources available on our website are:

1. The full baseline questionnaire: (http://www.chiwos.ca/wp-content/uploads/2014/08/CHIWOS-May-13-2014-En.pdf).
2. The *a priori* strategy for ensuring and determining the validity and reliability: (http://www.chiwos.ca/wp-content/uploads/2012/04/CHIWOS-Questionnaire-Development-Description_Feb-11-2014.pdf).

Visit us online at:

www.facebook.com/CHIWOS
www.twitter.com/CHIWOSresearch.

We aim to hasten knowledge creation regarding improving the health and wellbeing of women with HIV. We welcome collaborations regarding research ideas and questions, using the CHIWOS data and KT initiatives; please contact us via our website if you have interest.

## Supporting information

S1 Table Priority populations of harder-to-reach and underserved women with HIV for enrollment in CHIWOS. Explanation of harder-to-reach populations that were purposively recruited for the study.
(DOCX)

S2 Table CHIWOS questionnaire sections and themes and validated scales used. (DOCX)
