# Supplementary material for: Cohort profile: The Canadian HIV Women’s Sexual and Reproductive Health Cohort Study (CHIWOS)
Source: PLoS One. 2017 Sep 28;12(9):e0184708. doi: 10.1371/journal.pone.0184708 (PMC5619712; doi:10.1371/journal.pone.0184708)
Supplement: S1 Table — Explanation of harder-to-reach populations that were purposively recruited for the study. (DOCX) [file pone.0184708.s001.docx]

**S1 Table**

| **Population** | **Definition** | **Reason** |
| --- | --- | --- |
| **Trans women (n=54)** | Male-to-female trans persons including those in transition*.  *Transition: process trans people go through to overcome physical, legal and social barriers so they can express their self-identified gender. | Major gaps remain in knowledge about the epidemiology, management and response of women living with HIV across the varying geographic, social, and healthcare landscapes of Canada [5]. What is known is that HIV has tended to impact women who: are disproportionately affected by adverse social determinants of health, have personally or historically experienced colonization, including Indigenous women, are racialized within Canadian society, particularly those who identify as African, Caribbean or Black, women from other ethnic minority groups and immigrant women, use or inject drugs, are involved in sex work, have been incarcerated, are below the age of 30, and are LGBQQ2S and transgender [6]. These socially marginalized, harder-to-reach and underserved women living with HIV are known to have poorer engagement and retention in care and adverse health outcomes [5, 32].  CHIWOS aims to focus on harder-to-reach and underserved HIV-positive women as they often experience inequities in health care access, retention and outcomes. These health differences are unnecessary and unjust as well as avoidable as they are systemically related to social inequality and disadvantage.  Harder-to-reach and underserved communities have poorer health outcomes, use the emergency departments more frequently than other populations, and experience higher rates of hospitalization [6, 32]. By focusing on these populations in CHIWOS it will help assess the health care needs of harder-to-reach and underserved HIV-positive women which is critical to improving the engagement and retention in care of harder-to-reach and underserved HIV-positive women which is an important element in improving clinical health outcomes in Canada. |
| **Indigenous women (n=318)** | Women who have a historical continuity with pre-invasion and pre-colonial societies that developed on their territories (i.e. First Nations, Inuit or Métis), consider themselves distinct from other sectors of the societies now prevailing in those territories, or parts of them (also know as Aboriginal women). |  |
| **Caribbean (or West Indian) women (n=74)** | Women who are native inhabitants of the Caribbean region, or of Caribbean descent. |  |
| **Women from other ethnic minority groups (e.g. South Asia, Asia, Southeast Asia, Latin America) (n=120)** | Women who are native inhabitants or of descent from countries/continents in the Global South (excluding Africa and the Caribbean) such as Latin America, Asia, South Asia and the Middle East. |  |
| **LBQQ2S women (n=180)** | Women who identify their sexual orientation as lesbian, bi-sexual, queer, questioning and /or two-spirit*.  *****Lesbian: A female identified person who has romantic or sexual attractions primarily to people of the same gender or sex.  Bisexual: A person who may have romantic or sexual attractions to people of any gender or sex.  Queer: An umbrella term used proudly by some people to defy gender or sexual restrictions. This is also one way some people identify themselves as members of the lesbian, gay, bisexual, and/or trans communities or cultures. The term is not reclaimed by everyone and may be hurtful for some due to its historic derogatory connotation.  Questioning: A person who isn't certain if they are gay, lesbian, bisexual, or trans and are still trying to figure out how to identify themselves.    Two-spirit: A term based on interpretation of words used in different Indigenous cultures to refer to a person having both a male and female spirit. It can include Anglo/North American ideas of both sexual orientation and gender identity (i.e., both a gay cisgender man and a trans man could claim the identity of a two-spirit man). It also includes significant spiritual and cultural layers. |  |
| **Women who have injected or are injecting drugs (n=438)** | Women who have used, or are currently using injection drugs*.  *Injection drugs: are drugs that are introduced to the body with a hollow needle and a syringe, which is pierced through the skin into the body (usually intravenous, but also intramuscular or subcutaneous) such as heroin, cocaine, and methamphetamine. |  |
| **Women currently involved in sex work (n=82)** | Women who are currently involved in sex work*.  *Sex work: A job selling some form of sexual service, or the exchange of sexual services, or products for material compensation such as money, gifts, food, shelter, clothes, drugs and other goods. |  |
| **Women who are not accessing HIV care (n=77)** | Women who have not seen an HIV doctor/infectious diseases specialist in over one year. |  |
| **Young women (n=137)** | Women younger than 30 years of age. |  |
| **Overall not overlapping** | N = 540 |  |

CHIWOS, Canadian HIV Women’s Sexual and Reproductive Health Cohort Study; LBQQ2S, lesbian, bisexual, queer, questioning, or two-spirit*.*
