# Supplementary material for: Cohort profile: The Canadian HIV Women’s Sexual and Reproductive Health Cohort Study (CHIWOS)
Source: PLoS One. 2017 Sep 28;12(9):e0184708. doi: 10.1371/journal.pone.0184708 (PMC5619712; doi:10.1371/journal.pone.0184708)
Supplement: S2 Table — (DOCX) [file pone.0184708.s002.docx]

**S2 Table**

| **Section** | **Themes covered** | **Scales used** |
| --- | --- | --- |
| **Section 1: Demographic and Socio-Economic Status** | Age, gender, biological sex, sexual orientation, marital status, country of birth, ethnicity and/or racial background, immigrant status, education, employment, household gross annual income, sources of income, financial dependents, living status, and food security. | 1. Questions were developed by CHIWOS investigators. |
| **Section 2:**  **Medical and HIV Disease Information** | Body mass index, date of HIV diagnosis, mode of HIV transmission, access and delay to primary HIV medical care, use of and adherence to ART, antiretroviral types, CD4 count, viral load, co-infections, trans health, paediatric to adult HIV care transition. | 1. Questions were developed by CHIWOS investigators.  2. Antiretroviral therapy (ART) adherence was measured using the visual analogue scale (VAS) from the Medication Adherence Self-Report Inventory (MASRI) (Walsh et al., 2002). The VAS ask respondents to indicate on the scale how much medication they have taken in the last month with an answer ranging between 0% and 100% in 10% intervals. |
| **Section 3:**  **Health Care and Support Service Utilization** | Access and receipt of HIV medical care and other medical and support services, barriers /preference/diversity of HIV medical care and support services, type of HIV medical care provider, women-specific health and wellbeing knowledge by HIV medical care provider, characterization of women-centred HIV medical care. | 1. Questions were developed by CHIWOS investigators.  2. Questions regarding gender and language preference were modified from the Commonwealth Fund 2001 Health Care Quality Survey.  3. Questions listing services accessed and used were modified from A-Track (Public Health Agency of Canada) with content from the Canadian Community Health Survey (CCHS) (Government of Canada, Statistics Canada) and Special Projects of National Significance (SPNS)^;^ Cooperative Agreement Evaluation (Module 4A: Services needed and received: National evaluation by the measurement group) (U.S. Department of Health and Human Services, Health Resources and Services Administration, HIV/AIDS Bureau).  4. The Barriers to Care Scale (BACS) was used to identify the severity of the barriers of persons living with HIV in urban and rural areas experience (Heckman et al., 1998). Barriers were categorized into the following subscales: geography/distance (2 items), medical and psychological service (4 items), community stigma (2 items), and personal resources (4 items) for a total of 12 items. The answer options were: major problem, somewhat of a problem, very slight problem and no problem at all. |
| **Section 4:**  **Women`s Reproductive Health** | Fertility intention and behaviour, contraceptive use and pregnancy experience and history, use and access to pre-conception care and during pregnancy, access to gynaecological, cancer care and fertility treatment services, measures of menstruation and menopause, and screening of cervical dysplasia and cancer, osteoporosis, and breast cancer. | 1. Questions were developed by CHIWOS investigators. 2. The questions in this section were modified from the Stages of Reproductive Aging Workshop (STRAW) Recommendations and the ReSTAGE Collaboration Study of Women’s Health Across the Nation (SWAN) and the Women’s Interagency HIV Study (WHIS). 3. Questions regarding attitudes, feelings and experiences of current or most recent pregnancies were derived from the Pregnancy Risk Assessment Monitoring System (PRAMS) (Centers for Disease Control and Prevention). 4. A question from the Canadian Community Health Survey (CCHS) was used to determine folic acid use (Government of Canada, Statistics Canada). |
| **Section 5:**  **Stigma and Discrimination** | Stigma as it relates to HIV and gender, sex, and racial discrimination. | 1. HIV-related stigma is measured using the modified *Berger HIV-Stigma Scale* a ten-item measure of stigma perceived by Persons living with HIV (PLWH) based on four psychological factors (Berger et al., 2001; Wright et al., 2007). The 4 subscales included: personalized stigma, disclosure concerns, negative self-image, and concern with public attitudes about HIV. The items are rated on a 5-point scale ranging from ‘strongly agree’ to ‘strongly disagree’.  Additional concerns related to HIV disclosure were measured using the HIV/AIDS-Targeted Quality of Life Instrument (Holmes et al., 1998). The disclosure worries dimension consist of 5-items with a 5 option answer response ranging from ‘strongly agree’ and ‘strongly disagree’ with an extra question developed by CHIWOS investigators.  2. A modified version from the Everyday Discrimination Scale was used to measure experiences of racism, sexism and gender discrimination (Forman et al., 1997; Clark et al., 2004). Racial discrimination was measured through 8-items with a 6 option response answer scale ranging from ‘almost every day’ to ‘never’. Experiences of sexism were captured using two 8-item scales each with 6 response options ranging from ‘almost every day’ to ‘never’. One scale was specific to respondents self-identifying as a woman and another scale more broadly referring to discrimination related to gender.  3. A final scale in this section, captured acts of violence and discrimination related to gender identity using a 9-item measure with respondents answering one of 4 responses ranging from ‘many times’ to ‘never’. This scale was developed by the CHIWOS investigators. |
| **Section 6:**  **Substance Use** | Past and present substance use, hazardous alcohol use, treatment for addiction, and access and use of substance-related services. | 1. Questions were developed by CHIWOS investigators.  2. Alcohol Use was measured using 2 items from the Alcohol Use Disorders Identification Test (AUDIT) (Saunders et al., 1993; Babor et al., 2001) and an item providing information about binge drinking practices (D'Alessio, Baiocco, & Laghi, 2006; Vik, Tate, & Carrello, 2000). |
| **Section 7:**  **Violence and Abuse** | Violence and abuse during childhood and adulthood, location of violence or abuse such as country of origin or during residential school. | 1. Questions were developed by CHIWOS investigators. 2. Questions to screen for violence and abuse were modified from the Hurt, insult, Threaten, and Scream (HiTS) tool (Sherin, Sinacore, Li, Zitter, and Shakil, 1998). |
| **Section 8:**  **Women's Sexual Health** | Sexual behaviours, sexual satisfaction, sexual functioning and sexual negotiation. | 1. Questions were developed by CHIWOS investigators. 2. A modified 12-item Brief Index of Sexual Functioning for Women (BISF-W) will be used to measure levels of female sexual functioning and satisfaction. (Taylor et al., 1994). The scale has varied response options.      1. A modified 6-item Sexual Satisfaction Scale for Women (SSS-W) was used with a 5 answer response option; ‘Strongly agree’ to ‘Strongly disagree’ (Meston and Trapnell, 2005). |
| **Section 9:**  **Emotional Wellbeing, Resiliency, and Health Related Quality of Life** | Depression symptoms, post-traumatic stress disorder, quality of life, social support. | 1. Questions were developed by CHIWOS investigators. 2. The Center for Epidemiological Studies Depression Scale (CES-D) was used to screen for depressive symptoms (Radloff, 1977). This screen was used to characterize the ways a person might have felt or behaved during a past week from 10 items with 4 answer options ranging from ‘Rarely or none of the time (less than 1 day)’ to ‘Most or all of the time (5-7 days)’. 3. Post-traumatic stress disorder was measured using an abbreviated Post-traumatic Stress Disorder Checklist-Civilian Version (PCL-C) (Lang et al., 2005). This 6-item scale was used to assess problems or complaints resulting from stressful life events with 5 response options ranging from ‘Extremely’ to ‘Not at all’. 4. An abbreviated 4-item Medical Outcomes Study Social Support Survey (MOS-SSS) was used to measure different kinds of available social support ((Sherbourne and Stewart, 1991; Gjesfjeld et al., 2008). The survey contained four measures of social support: emotional/informational, tangible, affectionate, and positive social interaction. The 5 response options ranged between ‘None of the time’ and ‘All of the time’. 5. The 12-item Short Form Survey from the Rand Medical Outcomes Study version 2 (SF-12v2) was used to measure functional health and well-being from the patient’s perspectives (Ware et al., 2002; Quality Metric Incorporated). Physical and mental health composite scores are obtained from the 12-item survey which has diverse answer options for its questions. 6. The Brief Resiliency Scale is a 10-item scale measuring personal competence and acceptance of self and life was used. The 7 answer options range between ‘Strongly agree’ to ‘Strongly disagree’. |

CHIWOS, Canadian HIV Women’s Sexual and Reproductive Health Cohort Study.
